# Effect Anticipation Affects Perceptual, Cognitive, and Motor Phases of Response Preparation: Evidence from an Event-Related Potential (ERP) Study

**DOI:** 10.3389/fnhum.2016.00005

**Published:** 2016-01-26

**Authors:** Neil R. Harrison, Michael Ziessler

**Affiliations:** Department of Psychology, Liverpool Hope UniversityLiverpool, UK

**Keywords:** action effects, anticipation, event-related potential (ERP), response preparation, ideomotor

## Abstract

The anticipation of action effects is a basic process that can be observed even for key-pressing responses in a stimulus-response paradigm. In Ziessler et al.’s (2012) experiments participants first learned arbitrary effects of key-pressing responses. In the test phase an imperative stimulus determined the response, but participants withheld the response until a Go-stimulus appeared. Reaction times (RTs) were shorter if the Go-stimulus was compatible with the learned response effect. This is strong evidence that effect representations were activated during response planning. Here, we repeated the experiment using event-related potentials (ERPs), and we found that Go-stimulus locked ERPs depended on the compatibility relationship between the Go-stimulus and the response effect. In general, this supports the interpretation of the behavioral data. More specifically, differences in the ERPs between compatible and incompatible Go-stimuli were found for the early perceptual P1 component and the later frontal P2 component. P1 differences were found only in the second half of the experiment and for long stimulus onset asynchronies (SOAs) between imperative stimulus and Go-stimulus, i.e., when the effect was fully anticipated and the perceptual system was prepared for the effect-compatible Go-stimulus. P2 amplitudes, likely associated with evaluation and conflict detection, were larger when Go-stimulus and effect were incompatible; presumably, incompatibility increased the difficulty of effect anticipation. Onset of response-locked lateralized readiness potentials (R-LRPs) occurred earlier under incompatible conditions indicating extended motor processing. Together, these results strongly suggest that effect anticipation affects all (i.e., perceptual, cognitive, and motor) phases of response preparation.

## Introduction

There is now ample evidence that the performance of voluntary actions includes the anticipation of action effects. First of all, the anticipated effect stands for the desired effect of the action. From a theoretical point of view, early theories of motor control considered the anticipation of the effect as a prerequisite to performance of the action. In a first step, randomly executed movements are associated with their environmental effects. In a second step, the activation of the effects in memory will lead to the reactivation of the movements that lead to this effect (e.g., Herbart, 1824; Lotze, 1852; Harleß, 1861; Münsterberg, 1888; James, 1890). In more modern terms, it has been argued that actions are represented in memory by their sensory effects (Greenwald, 1970; Prinz, 1983, 1997; Hommel et al., 2001; Hommel, 2009). Whereas those theories assume that effect anticipation is crucial for the selection of voluntary actions, other theoretical accounts argue that anticipation of effects is part of the control processes to plan and to execute the action. For example, Schmidt’s (1975, 1988) Schema Theory assumes that action effects are anticipated to allow an internal test to assess if the planned action will lead to the desired effect and to monitor the execution of the action by comparing the anticipated effects with the actual effects. Similarly, forward models describe the anticipation of effects for a planned action (Davidson and Wolpert, 2005; Wolpert and Flanagan, 2009). Thus, effect anticipation is considered as an important component for error detection and correction.

Compared to the theoretical underpinning, the empirical evidence for the different functions of effect codes in the selection, preparation and execution of motor actions remains limited. Many experiments, using a range of different paradigms, have clearly demonstrated that effect codes are activated in the course of action planning and preparation, but the exact nature of the activation of effect codes and their function in the process of action preparation is still under discussion (see Nattkemper et al., 2010; Shin et al., 2010). One of the first paradigms to prove the involvement of effect anticipation in the control of motor actions was based on the two-step model of James (1890) ideomotor principle. Elsner and Hommel (2001) let their participants execute two key-presses, and each key-press was followed by a particular tone. In so doing, participants were assumed to acquire response-effect relations. The tones were then used in the subsequent test phase as imperative stimuli. Participants responded faster to the stimuli in the test phase, if the stimuli were the former effects of the responses. Elsner and Hommel (2001) took this as evidence for the ideomotor principle, assuming that the effects would automatically activate the responses that produce those effects. Even though there has been some critical discussion about whether the experiments following this paradigm really show that it is the pre-activation of effect codes that leads to the selection of the responses (Ziessler and Nattkemper, 2011; Cox and Hasselman, 2013), this is still an influential paradigm. A significant limitation of the paradigm is that the response effect is physically presented in the test phase before the execution of the response. The ecological validity of this paradigm is therefore limited because under normal circumstances the physical presence of the effect would indicate that the response had been successfully completed. We cannot exclude that under those conditions the effect information is only used for the control of the response because the effects were presented as stimuli.

A similar argument can be applied to a second paradigm in which effect stimuli were presented during the preparation of the response. Ziessler et al. (2002, 2004); Ziessler and Nattkemper (2011) used a flanker paradigm (Eriksen and Eriksen, 1974) to investigate the integration of effect anticipation in the preparation of motor responses. In their experiments key-presses were followed by letters appearing on the computer screen. In the acquisition phase, participants learned which key-press was followed by which of the letters. In the subsequent test phase the imperative stimulus letters were accompanied by flanking letters which could be either the effects of the correct response, effects of a different response, or a neutral letter. The main result revealed that the responses were facilitated if the flankers were the effects of the required response. However, again we cannot be sure if effect codes would also be activated if they were not physically present in the form of the flanker stimuli.

More convincing paradigms are those in which the effects themselves are not physically presented, but for which we can conclude that effect anticipation indeed took place. For example, Kunde et al. (2002) provided participants with a response cue before presentation of the imperative stimulus. The cue allowed the participants to prepare the response. In the critical trials the imperative stimulus did not require the pre-cued response. The switch to the new response was faster if the new response produced the effect tone of the originally prepared response. Thus, it can be concluded that anticipation of the effects was at least part of the preparation of the response, but we do not know what exactly the anticipation of the effects contributed to the preparation of the responses.

Similarly, effects of the compatibility between the responses and their effects are evidence for the activation of effect codes in the course of response preparation. In those experiments responses are facilitated if there is an overlap between response features and effect features (Greenwald, 1970). For example, a left response is facilitated if the effect of the response appears on the left side or a low intensity response is facilitated if there is also a low intensity effect compared to conditions in which left responses would be followed by right effects or low intensity responses by high intensity effects (Kunde, 2001, 2003; Kunde et al., 2004).

Using a quite different paradigm, also Kühn et al. (2010) showed that the preparation of motor responses led to an activation of effect codes independently of the physical presence of the effect stimuli. In their experiment different key-presses produced houses or faces on the computer screen during the acquisition phase of the experiment. In the test phase those response effects were no longer presented, and participants only pressed the keys according to their free choice. The authors recorded the activations in different regions of the brain using fMRI. They found that depending on the selected key-press there was activation in the fusiform area if the former effect of the key-press was the presentation of a face and activation in the parahippocampal area if the former effect was a house. In other words, even though the effects were not physically present in the test phase, the preparation and execution of the responses activated the same areas in the brain that would be activated by the presentation of the original response effects.

Whereas the different paradigms described above together support the notion that the preparation of motor responses includes the activation of their effects in memory, most of these experiments do not allow convincing conclusions to be drawn about the nature of effect anticipation and function of the effects codes for the preparation of the motor responses. In the framework of the ideomotor principle (James, 1890) and the Theory of Event Coding (Hommel et al., 2001), Elsner and Hommel (2001) assumed that the activation of effect codes would lead to the selection of the responses. Therefore, temporally the anticipation of the effects should precede response selection. However, Ziessler and Nattkemper (2011) could not find a facilitation of the responses if effect stimuli were presented in advance of the imperative stimulus. But if the effect stimuli were presented with or shortly after the imperative stimulus, facilitation of the response was observed. This is in line with the idea that effects are anticipated in the course of response preparation to perform internal tests of the prepared response before its execution and to allow the monitoring of response execution and error detection by comparing the actual with the anticipated effects. Desantis et al. (2014) identified a critical time window from about 220 ms before onset of a motor action until 280 ms after execution. Within this time period the discrimination of stimuli that corresponded to the anticipated effects was improved. This finding supports Ziessler and Nattkemper (2011) view that effect anticipation depends on the selected motor response and is used to enable the monitoring of response execution. In the time window around the execution of the response the sensory system is prepared to process the effect stimulus. Additional support for this idea comes from Hughes and Waszak (2011) who analyzed event-related potentials (ERPs) evoked by the presentation of effect stimuli after response execution. If the presented stimuli were response effects they found an increased visual P1 component which indicated enhanced visual processing of the stimulus.

Wirth et al. (2015) investigated the stage at which response-effect anticipation takes place in a Psychological Refractory Period (PRP) paradigm (Pashler, 1984). The critical independent variable was response-effect compatibility. They found that the effect of response-effect compatibility was due to processes within or after the central bottleneck, i.e., the effect seemed to be caused by processes of response selection or subsequent mechanisms. Other authors focused more on the nature of the anticipated effect codes. For instance, in a review article Waszak et al. (2012) came to the conclusion that the preparation of an action pre-activates the sensory networks that represent the perceptual consequences of the action effect. In other words, the anticipation of the effects activates the perceptual areas in the brain that would also be active if the effects were actually perceived (Kühn et al., 2010; Roussel et al., 2013). Roussel et al. (2013), see also Hughes et al. (2013) argued that the pre-activation of the sensory networks would led to sensory attenuation for the predicted action effects. In line with this idea, Cardoso-Leite et al. (2010) observed reduced sensitivity for effect-congruent visual patterns in a detection task where Gabor patterns were presented with low contrast after a motor response. If the pattern was the learned effect of the motor response, participants showed reduced performance in detecting the orientation of the pattern. Stenner et al. (2015) assumed a relationship between sensory attenuation and the sense of agency; stronger sensory attenuation seems to lead to a stronger subjective sense of agency. Stimuli that corresponded to the anticipation of the actor required less perceptual processing as compared to non-corresponding stimuli. Mismatch between the anticipated and the actual effects triggered processes of error handling. In an EEG study, Waszak and Herwig (2007) found larger P3a amplitudes for deviant (i.e., unanticipated) effect stimuli. As Adachi et al. (2007) pointed out, the enhanced P3a component in the case of mismatch between the anticipated stimulus and the actual stimulus is not just due to the unexpectedness of the stimulus; the P3a enhancement is stronger if the stimulus was anticipated as an effect of a selected action.

So far the reviewed research shows that the preparation of motor actions includes the anticipation of effects which seems to be based on an activation of sensory networks. Codes of the anticipated effects are activated in memory as if the effects were actually perceived. In an attempt to analyze the temporal dynamics and the nature of effect anticipation in more detail, Nikolaev et al. (2008) adapted Ziessler and Nattkemper’s (2011) flanker experiment and recorded the ERPs elicited by the flanking stimuli. As described above, in the experiment the imperative stimulus determining the response was flanked by learned effects of the required response or by other stimuli. Presentation of the effects facilitated the response, in particular if the flanking stimuli appeared together with or shortly after the imperative stimulus. Significant differences in ERPs were found for the early visual P1, the frontal P2 and the response-locked lateralized readiness potential (R-LRP) components. If the flankers were the correct effect of the response under preparation, the P1 component was enhanced. This can be interpreted as reflecting a facilitation of the perceptual processing of the effect stimulus by a top-down tuning of the perceptual system for the processing of the effects. The later frontal P2 showed higher amplitudes if the flanker stimuli were not the effects of the response under preparation. Assuming that response preparation includes the anticipation of the effects, the non-effects were unexpected stimuli appearing in the context of the response. The higher P2 amplitudes indicated the mismatch between the expectations and the actual stimuli. Finally, for effect flankers the time delay between the onset of the R-LRPs and the onset of the response was shorter than for non-effect flankers. This is an indication that the mismatch of the flanking stimuli with the anticipated effects led to extended motor processing. It is important to note that the differences described above were mainly found after sufficient practice (i.e., in the second half of the experiment) and for the longer stimulus onset asynchronies (SOAs) between the presentation of the imperative stimulus and the flankers. Nikolaev et al. (2008) argued that participants first had to learn the response-effect relations and that they needed time to anticipate the response effect after response selection. The points in time at which significant differences in the P1, P2 and LRPs were found support the view that effect codes were used for the internal test of an already selected motor response and for monitoring the execution of the response.

With the present study we wanted to apply a similar method to another experimental paradigm introduced by Ziessler et al. (2012). As discussed earlier, a criticism to the flanker experiments by Ziessler et al. (2004) and Ziessler and Nattkemper (2011) is that the effects were physically presented in the experiment. Even though the results support the assumption that effect codes were activated in the course of response preparation, we cannot exclude the possibility that this only occurred because the participants could expect the physical presentation of the effect stimuli before the execution of the responses. Importantly, Ziessler et al. (2012) avoided this problem by presenting other stimuli during the preparation of the response that were either compatible or incompatible with the later effects of the response. In their Experiment 3, participants learned that key-presses with the index and middle fingers of both hands were followed by pictures of particular hand postures on the computer screen. In the test phase of the experiment, participants were asked to prepare the response determined by an imperative stimulus (color of a square), but to withhold it until a Go-stimulus was presented. The Go-stimuli were objects that each fitted with one of the different hand postures used as response effects. On appearance of the Go-stimulus participants should execute the required response. Responses were faster if the Go-stimulus was compatible with the hand posture that could be anticipated as the effect of the required response. The authors argued that the compatibility between Go-stimuli and response effects could only affect the response times (RTs) if codes of the response effects were already activated when the Go-stimuli appeared.

In the present study we repeated Ziessler et al.’s (2012) experiment while simultaneously collecting EEG data, and we analyzed ERPs elicited by the Go-stimuli, and motor-related neural activity. The four key-presses with the left and right index and middle fingers were followed by a hand in the posture of holding a coffee mug, a computer mouse, a pen and a spoon as effects. In the test phase green, red, orange and blue squares determined the response. Participants were instructed to only execute the response on presentation of a Go-stimulus after a variable SOA between the imperative stimulus and Go-stimulus. The SOA variation was used to present the Go-signal at different stages of response preparation. Pictures of a coffee mug, computer mouse, pen and spoon were used as Go-stimuli. The Go-stimulus was not indicative of the required response. For each response two Go-stimuli were used, one of them compatible with the learned effect and the other incompatible. Both Go-stimuli appeared with the same frequency for the respective response. Thus, participants could not develop expectations about a particular Go-stimulus related to the response.

The original experiment (Ziessler et al., 2012) showed that the compatibility between Go-stimulus and response effect influenced the RTs. In the current experiment we were mainly interested in the ERPs evoked by the Go-stimuli. Significant differences in the ERPs could provide us with additional information about the temporal dynamics and the nature of effect anticipation. ERPs related to early visual processing such as the P1 component would support the view that effect anticipation consists of a pre-activation of sensory networks, which could affect the early visual processing of the Go-stimuli. However, if the Go-stimuli interacted with codes of the anticipated effects only at a more conceptual level, no differences in the early visual potentials would be expected. Instead it would be more likely to find differences in later potentials reflecting deviance from expectations, for example in the fronto-central P3a component. Furthermore, if effect anticipation depended on the stage of response preparation, ERPs to the Go-stimulus should depend on the SOA between imperative stimulus and Go-stimulus. ERP differences at early stages of response preparation would indicate that effect codes were already active with the selection of the response. However, if the effect anticipation would follow response selection it would be more likely to find differences in the ERPs to the Go-stimuli only at longer SOAs.

## Materials and Methods

### Participants

Eleven adults participated in the experiment (mean age = 37.4 years, range: 21–57 years, *SD* = 11.6; 4 women). Two of the participants were dominantly left handed, the others were dominantly right handed. Data from one participant was excluded from all analyses due to excessive artifacts in the EEG recording. All participants gave informed written consent in accordance with the Declaration of Helsinki, and the study was carried out in accordance with the recommendations of the Psychology Research Ethics Committee of Liverpool Hope University.

### Materials and Apparatus

**Figure 1:**
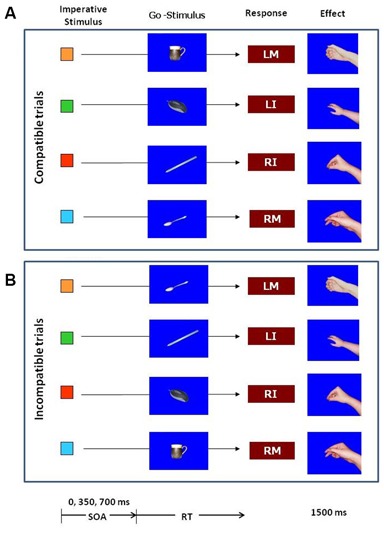
**Stimuli and procedure in the test phase of the experiment.** The color of the imperative stimulus determined the response. After 0, 350 or 700 ms one of the objects was presented as a Go–stimulus in 82% of the trials. In the remaining 18% of trials a picture of a hammer (not shown in the figure) served as NoGo stimulus. Responses were key-presses with the left middle finger (LM), left index finger (LI), right index finger (RI), or right middle finger (RM). After the key-press the effect was presented for 1500 ms. The effects were pictures of hand postures. The upper part **(A)** illustrates the trials in which the Go-stimulus was compatible with the response effects, the lower part **(B)** illustrates the incompatible trials.

The experiment was controlled by a standard PC using E-Prime software (Psychology Software Tools Inc., PA, USA). In the first part of the experiment, participants learned the effects of their responses by pressing a key on the PC keyboard with the index or middle fingers of the left and right hand. The fingers were placed on the keys “Z”, “X”, “N” and “M” on the QWERTY keyboard. The stimulus to initiate a key press was the letter “O” presented in the middle of the screen. Response effects were pictures of a right hand in the posture as if holding a coffee mug, a computer mouse, a tea spoon or a pen (Figure 1). The pictures only showed the hand without the object. In the test phase the pictures of the corresponding objects were introduced as Go-stimuli. There was a picture of a coffee mug, a computer mouse, a tea spoon and a pen. A picture of a hammer was used as NoGo stimulus. All pictures were presented in the middle of the screen on blue background within a visual angel of about 10°. Also in the test phase small squares of 1 × 1 cm colored in blue, red green or orange, assigned in order to the left middle finger, left index, finger, right index finger, and right middle finger were introduced as imperative stimuli.

### Design and Procedure

The experiment consisted of an acquisition phase and a test phase. The purpose of the acquisition phase was that participants would learn arbitrary effects for the four key presses. In a free-choice task participants performed one of the four key presses after an “O” appeared in the middle of the screen. Participants were instructed that they could use any key, but they should try to make sure that all keys were used approximately the same number of times. Key presses with the left middle finger were immediately followed by a hand in the posture of holding a computer mouse. A key press with the left index finger generated a hand in the posture of holding a coffee mug. The right hand key presses produced a hand in the posture of holding a tea spoon for the index finger and of holding a pen for the middle finger. The effect pictures remained on the screen for 1500 ms. Following a 500 ms blank screen the next trial started with the presentation of an “O”. All together there were 100 acquisition trials with about 25 trials for each response. According to experiments reported in the literature, the free-choice task should provide optimal conditions for response-effect learning (Elsner and Hommel, 2001; Herwig et al., 2007; Pfister et al., 2010).

On completion of the acquisition phase participants were instructed regarding the stimulus-response assignment for the forced-choice task in the test phase, i.e., they were shown on the computer screen which color was allocated to which of the four responses. The presentation of the instruction was self-paced so that participants could memorize the stimulus-response assignment as long as they wished. In the test phase (see Figure 1), each trial started with the presentation of a fixation cross in the middle of the screen. After 1000 ms the fixation cross was replaced by a blue, red, green or orange square. The color of the square determined the response as defined by the stimulus-response assignment. Participants were instructed to prepare the corresponding key press, but not to execute until a Go-stimulus appeared. The Go-stimulus appeared behind the little square so that the imperative stimulus remained on the screen. On appearance of the Go-stimulus participants were asked to execute the prepared response as fast as possible. The response terminated the presentation of the imperative and the Go-stimulus and the response effect appeared on the screen for 1500 ms, provided the response was correct. If the response was erroneous, the word “incorrect” was presented instead of the effect. There was a maximum time limit for each response of 3000 ms. If the NoGo signal (the picture of a hammer) appeared no response should be made. The NoGo stimulus remained on the screen for 2000 ms. If a response was made despite the presentation of the NoGo stimulus (i.e., a false alarm), the participants were reminded not to respond in that case. All trials were separated by a blank screen of 500 ms duration.

The NoGo were important to check if participants followed the instructions. Responses in NoGo trials (the false alarms) could only be avoided if participants waited for the presentation of the Go-stimulus with the execution of the response. Therefore, as in Ziessler et al. (2012) participants with false-alarm rates higher than 25% of the NoGo trials will be excluded from the analysis.

There were two independent variables: the first independent variable was the compatibility between the Go-stimulus and the effect of the response. In half of the Go trials the Go-stimulus was compatible with the response effect, in the other half incompatible. For example, the blue square the participants required a key press with the middle finger of the left hand. The effect of this response is the hand in the posture of holding a computer mouse. Participants were instructed to prepare the response and execute it as soon as a Go-stimulus was presented. The compatible Go-stimulus in this case is the picture of the computer mouse, whereas the picture of a spoon would be incompatible with the response effect. To avoid the Go-stimulus priming a particular response, we did not use the remaining three object pictures as incompatible Go-stimulus for each of the responses, but only one of them. Thus, each of the four Go-stimuli was used for one response as compatible Go-stimulus and for another response as incompatible Go-stimulus with a probability of 50%. The second independent variable was the SOA between the onset of the imperative stimulus and the onset of the Go-stimulus. Three SOAs were used: 0, 350, and 700 ms. Thus, depending on the SOA, the Go-stimulus appeared at different stages of the preparation of the response. The NoGo stimulus was always presented with the 700 ms SOA.

The complete test phase consisted of 552 trials. One hundred twenty trials were NoGo trials and 432 were Go trials equally divided over the four responses, compatible and incompatible Go-stimuli, and the three SOAs. The test phase was split in six blocks of trials to give participants a rest from time to time. After each block participants received feedback regarding their mean RTs, the number of errors and the number of false alarms. The experiment including both phases lasted about 1 h.

### EEG Data Acquisition

EEG data was recorded from 64 electrodes using an Active Two amplifier system (BioSemi, Amsterdam, Netherlands). Electrodes were positioned according to the extended 10–20 system (Nuwer et al., 1998). Four additional leads were placed above and below the left eye and on the outer canthi of the left and right eyes, to record the vertical electrooculogram (VEOG) and horizontal electrooculogram (HEOG). EEG from all channels was acquired with respect to the common mode sense (CMS) electrode at a sampling rate of 512 Hz.

### Go-Stimulus Locked ERP Analysis

The continuous EEG was divided into epochs offline, beginning 100 ms prior to the Go-stimulus and ending 800 ms after the Go-stimulus. The averages were digitally filtered (second-order zero-phase-lag bandpass filter, 0.5–25 Hz). ERP amplitudes were aligned to a 100 ms pre-stimulus baseline period. EEG artifacts were rejected using the SCADS procedure with standard parameters (Junghöfer et al., 2000). This procedure first detects individual channel artifacts, then transforms the data to average reference and then detects global artifacts. Epochs that contained more than 10 unreliable electrodes were excluded from analysis on the basis of the distribution of their amplitude, standard deviation and gradient. For the remaining epochs data from artifact-contaminated electrodes was replaced by a statistically weighted spherical interpolation using the complete set of channels. Regarding the spatial distribution of the approximated electrodes, it was ensured that the rejected channels were not localized within one scalp region, as this would make interpolation for this area unreliable. Therefore the standard deviation of the spherical splines used for approximation was computed for each epoch and epochs that represented outliers from this distribution were rejected. Across all participants and all conditions the procedure rejected an average of 27.7% of epochs as contaminated. On average, the number of epochs analyzed per participant in the first half of the experiment was 74.4 in the compatible conditions, 77.9 in the incompatible conditions, and 36.5 in the No-Go condition. In the second half of the experiment, the average number of epochs analyzed per participant was 78.6 in the compatible conditions, 79.2 in the incompatible conditions, and 35.9 in the No-Go condition.

### Statistical Analysis: Go-Stimulus Locked ERPs

The P1, N1, P2, and N2 ERP components of the Go-stimulus locked waveforms were analyzed at electrode sites selected on the basis of inspection of the grand averaged waveforms, inspection of the topographical maps, and guided by previous studies. We analyzed the P1 and N1 components at left (PO3, PO7, P7) and right (PO4, PO8, P8) electrode positions, where these components were largest, as indicated in the topographic maps showing a bilateral pattern of activation over occipito-parietal electrodes (see Figures 3, 4). These electrode locations corresponded closely with those reported in previous studies (e.g., Störmer et al., 2009; Krämer et al., 2013). For the P1 we used a time-window from 90 to 130 ms (i.e., ±20 ms around the peak at 110 ms) and for the N1 we employed a time-window from 150 to 190 ms (i.e., ±20 ms around the peak at 170 ms). These time-windows are identical to those reported in several previous ERP studies investigating the visual P1 and N1 components (e.g., Eimer and van Velzen, 2005; Lloyd-Jones et al., 2012). Mean amplitudes within the electrode clusters were analyzed using repeated measures ANOVA with the factors Compatibility (compatible, incompatible), Practice (first half, second half), SOA (0, 350, 700), and Laterality (left, right).

The P2 and N2 components were analyzed at two electrode clusters over fronto-central scalp (fronto-central cluster: FCz, Cz, C1, C2; frontal cluster: AFz, Fz, F1, F2), based on the midline fronto-central scalp distribution of these components (see Figures 6, 7), in line with previous studies (e.g., Sheng et al., 2015). For the P2 components, mean amplitudes were calculated between 140–200 ms (i.e., ±30 ms around the peak at 170 ms), which corresponds closely to the time-window used in previous ERP studies (e.g., Niedeggen et al., 2014; Sheng et al., 2015), and for the N2 component mean amplitudes were calculated between 220–280 ms (i.e., ±30 ms around the peak at 250 ms), in agreement with previous studies (e.g., Sheng et al., 2015). Mean amplitudes for the electrode clusters were analyzed using repeated measures ANOVA with the factors Compatibility (compatible, incompatible), Practice (first half, second half), SOA (0, 350, 700), and Scalp Region (fronto-central, frontal).

### Statistical Analysis: LRPs

LRPs were calculated using the standard procedure (Coles, 1989). We subtracted the signal of the C3 electrode from the C4 electrode signal to obtain the activity related to the left-hand response and subtracted the C4 signal from the C3 for the right-hand response. The averaged right and left difference waves constituted the LRP (c.f. Coles, 1989). In short:

LRP = [lefthand(C4-C3)+righthand(C3-C4)]/2

Stimulus-locked LRPs (S-LRP) were obtained using a 1200 ms epoch with a 200 ms pre-stimulus baseline. R-LRPs were derived over a 1300 ms epoch with a −1100 to −500 ms baseline and a 200 ms post-response interval. The averages were digitally filtered (second-order zero-phase-lag bandpass filter, 0.01–6 Hz). Artifact detection and correction, and re-referencing were the same as described for the Go-stimulus locked ERPs. S-LRPs and R-LRPs were obtained for compatible and incompatible conditions, averaged over SOAs and first and second half, to ensure a sufficient number of trials to compute reliable LRP waveforms (c.f. Eimer, 1998).

For statistical analysis of LRP onset, we applied a jackknife-based method (Miller et al., 1998). For the compatible and incompatible conditions, 10 subsamples of grand average LRPs were computed by omitting from each subsample the LRP data of a different participant. In each subsample we determined the LRP onset as the time point at which a threshold of −1 mV was exceeded. An absolute criterion as threshold is suggested by Miller et al. (1998) in the case of different peak amplitudes across conditions. Subsample onset values were analyzed with a repeated-measures ANOVA, and then correction was applied to the *F*-value as proposed by Ulrich and Miller (2001): *F*_c_ = *F*/(*n* − 1)^2^, where *F*_c_ denotes the corrected *F*-value and *n* the number of participants. For R-LRPs, R-LRP mean amplitudes were calculated during the 400 ms prior to response and were compared between compatible and incompatible conditions. For S-LRPs, S-LRP mean amplitudes were derived between 400–800 ms post-stimulus onset.

### Statistical Analysis: Behavioral Data

Reaction times (RTs) for correct responses were analyzed using a three-way repeated-measures ANOVA. The first factor (“Compatibility”) was the compatibility relationship between the Go-stimulus and the response effect (compatible vs. incompatible). The second factor was the SOA (0, 350, 700 ms). In addition to these independent variables we also took a practice factor (“Practice”) into account. To have sufficient data for each condition, the experiment was split into two halves and we tested the first half against the second half.

## Results

### Analysis of the Behavioral Data

The only purpose of the acquisition phase was to make the participants familiar with the response effects. On average the participants followed the instruction to use all responses with approximately the same frequency. The left index finger was used on average 24 times by each participant, the left middle finger 26 times and the two responses with the right hand 25 times each. Thus, participants had approximately equal experience with all response-effect relations.

With regards to the research question only the data from the test phase are of interest. In a first step, we analyzed the number of false alarms for each participant, i.e., the number of responses in NoGo trials. This is an important indicator as to whether the participants followed the instruction to prepare the responses but to withhold them until the Go-stimulus was presented. High false alarm rates would indicate that the participants did not pay attention to the Go and NoGo stimuli and consequently the different types of Go-stimuli could not affect the preparation of the responses. Participants made on average 6.8% of false alarms with a standard deviation *(SD)* of 3.2. The highest individual false alarm rate was 13%. This indicates that all participants in most of the trials differentiated between the Go-stimuli and the NoGo stimulus correctly. None of the participants was excluded from further analysis because of too many false alarms.

For the analysis of the RTs only correct responses were taken into account. On average 2.06% of the responses were errors (*SD* = 0.97). Furthermore, outliers were excluded from the analysis. This applied to RTs deviating more than three standard deviations from the mean RT for each SOA within each participant. As outliers 1.66% of the data (*SD* = 0.46) were discarded.

The remaining 96.28% of the data were averaged over the ten participants for each experimental condition. The individual mean values were subjected to a three-way repeated-measures ANOVA with the factors Compatibility (compatible, incompatible), SOA (0, 350, 700 ms), and Practice (first half, second half). The sphericity assumption was tested using Mauchly’s test of Sphericity. The test indicated that sphericity could be assumed.

**Figure 2:**
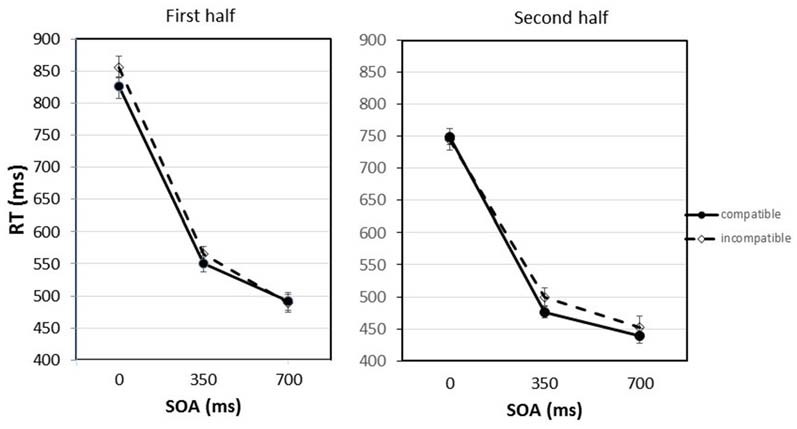
**Response times (RTs) for compatible and incompatible trials depending on the stimulus onset asynchrony (SOA) between the imperative stimulus and the Go-stimulus separated for the first and second half of the experiment.** Error bars represent the standard error of the means *(SEM)* calculated for the within-participant design following the procedure suggested by Cousineau (2005).

Figure 2 shows the mean RTs for compatible and incompatible trials depending on the SOA between the imperative stimulus and the Go-stimulus, separated for the first and second half of the experiment.

The statistical analysis revealed that the practice effect was significant, *F*_(1,9)_ = 16.28, *p* = 0.003. The average RTs decreased from the first to the second half of the experiment from 630 ms to 560 ms. Also the main effect of SOA was significant, *F*_(2,18)_ = 248.29, *p* < 0.001. If the Go-stimulus appeared together with the imperative stimulus (SOA = 0 ms), mean RTs amounted to 793 ms. With the longer SOAs, RTs became shorter. After the 700 ms SOA a mean RT of 468 ms was observed. Pairwise comparisons applying the Bonferroni correction confirmed that the differences between all SOAs were significant. Most importantly, there was also a significant main effect of compatibility, *F*_(1,9)_ = 11.20, *p* = 0.009. The RTs were on average 12 ms shorter if the Go-stimulus was compatible with the response effect that was presented after the response had been executed. This compatibility effect interacted with SOA and practice, *F*_(2,18)_ = 4.50, *p* = 0.026. In the first half of the experiment differences between compatible and incompatible trials were mainly observed at the short and middle SOA; in the second half of the experiment the compatibility effect appeared at the middle and long SOA. Planned comparisons (paired-samples *t*-tests) revealed that the difference between compatible and incompatible trials was significant in the first half of the experiment for the 0 ms SOA, *t*_(9)_ = 2.57, *p* = 0.030. For the 350 ms SOA the compatibility effect just failed the significance level, *t*_(9)_ = 2.08, *p* = 0.068. In the second half of the experiment only at the 350 ms SOA there was a trend for the compatibility effect to be significant, *t*_(9)_ = 1.90, *p* = 0.090. It should be noted that the planned comparisons between compatible and incompatible trials at single SOAs only include a limited amount of data. To secure statistical significance for a very small effect at this level more statistical power would be required. Apart from that, only the interaction between practice and SOA was significant, *F*_(2,18)_ = 6.55, *p* = 0.007. RTs following the longer SOAs benefited more from practice than RTs following the shorter SOAs. The interactions between compatibility and practice and between compatibility and SOA were not significant, *F*s < 1.

To exclude the possibility that the compatibility effect might be caused by a speed-accuracy trade off, the percentage of errors in the compatible and incompatible trials was tested against each other. In contrast to a speed-accuracy trade off, there was a very small tendency for lower error rates in compatible trials (2.0%) (the faster responses) as compared to incompatible trials (2.3%) (the slower responses), but this difference was not significant, *t*_(9)_ = 0.67, *p* = 0.519.

### Go-Stimulus Locked ERPs

The analysis of the Go-stimulus locked ERPs was focused on the P1, N1, P2 and N2 components. All components were analyzed with the following factors: Compatibility (compatible, incompatible), reflecting the compatibility between Go-stimulus and response effect; Practice (first half, second half of the experiment); and SOA (0, 350, 700), reflecting the SOA between the imperative stimulus and the Go-stimulus. For the P1 and N1 components we included the factor Laterality (left occipito-parietal region, right occipito-parietal region), and for the P2 and N2 components we included Scalp Region (fronto-central, frontal).

#### P1

**Figure 3:**
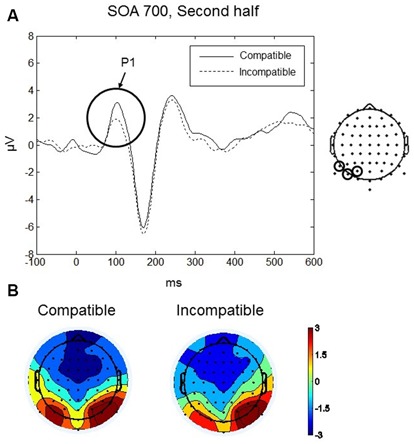
**Go-stimulus locked responses for the P1 component. (A)** Event-related potentials (ERPs) to compatible (solid line) and incompatible (dashed line) conditions are shown over the left occipito-parietal scalp region. Between 90–130 ms amplitudes to compatible signals were more positive than amplitudes to incompatible signals. **(B)** Scalp topographic maps at 110 ms after onset of the Go-stimulus for compatible (left) and incompatible (right) conditions. In this and the following figures, the electrodes at which the reported ERPs were observed are indicated on the map on the right.

P1 was maximal over bilateral occipito-parietal electrodes at around 110 ms (see Figures 3A,B). Analysis focussed on the scalp region where the amplitude was greatest, using a cluster of three occipito-parietal electrodes on the left (PO3, PO7, P7), and three on the right (PO4, PO8, P8). Between 90–130 ms we found a significant four-way interaction between Compatibility, Practice, SOA and Laterality: *F*_(2,18)_ = 6.25, *p* < 0.009. No other main effects or interactions were significant. To interpret the four-way interaction we performed two three-way ANOVAs with factors SOA, Compatibility, and Laterality, separately for each level of the factor Practice (first half and second half). In the first half of the experiment, there were no significant main effects or interactions (all *p*s > 0.09). In the second half, we found a significant interaction between SOA, Compatibility, and Laterality, *F*_(2,18)_ = 3.92, *p* = 0.039. *Post hoc t*-tests revealed a significant difference between compatible and incompatible conditions in the left occipito-parietal region (*t*_(9)_ = 4.43, *p* = 0.002) at the 700 ms SOA. For the 700 ms SOA in the second half of the experiment the P1 amplitude for the compatible condition was greater (mean = 2.51, *SD* = 2.11 μV) than the amplitude for the incompatible condition (mean = 1.39, *SD* = 1.63 μV; Figure 3A). There was no difference between compatible and incompatible conditions in the right occipito-parietal region (*p* = 0.838), and there were no differences between compatible and incompatible conditions at 0 ms SOA or at 350 ms SOA (all *p*s > 0.11).

#### N1

**Figure 4:**
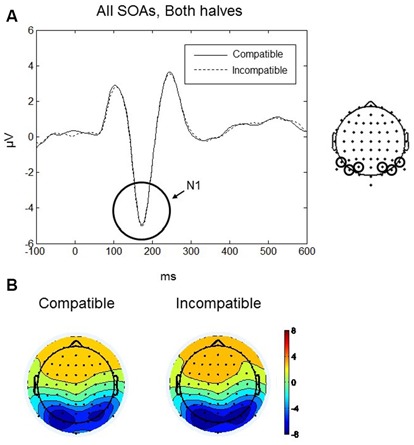
**Go-stimulus locked responses for the N1 component. (A)** ERPs to compatible (solid line) and incompatible (dashed line) conditions are plotted over occipito-parietal scalp regions. Between 150–190 ms there were no differences between compatible and incompatible conditions. **(B)** Scalp topographic maps at 170 ms after onset of the Go-stimulus for compatible (left) and incompatible (right) conditions.

The maximum amplitude for N1 was found at 170 ms, and the scalp distribution showed greatest activity bilaterally over occipito-parietal electrodes (Figures 4A,B). Mean amplitudes within the left and right occipito-parietal electrode clusters were analyzed between 150–190 ms. No main effects or interactions reached significance. Thus, the N1 did not differentiate between compatible and incompatible Go-stimuli, neither in the first nor in the second half of the experiment or between the different SOAs. Figure 4A shows the N1 component evoked by compatible and incompatible Go-stimuli averaged over all SOAs and both halves of the experiment.

**Figure 5:**
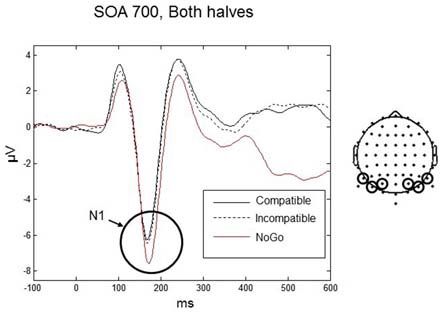
**Responses for the N1 component for the Go-stimuli (black solid and black dashed lines) and the NoGo stimuli (red line) over occipito-parietal electrodes.** The amplitude for the NoGo stimuli was enhanced compared to the Go-stimuli, reflecting discrimination between Go and NoGo signals. Similar amplitudes for the compatible and incompatible Go conditions confirms the pattern for all SOAs shown in Figure 4.

The N1 is often described as a component related to early stimulus discrimination processes (e.g., Vogel and Luck, 2000). In the present experiment, participants did not have to discriminate between compatible and incompatible Go-stimuli. However, they had to discriminate between Go-stimuli and NoGo stimuli. Therefore, in an additional N1 analysis we included the NoGo condition. Data were analyzed only for the 700 ms SOA (as only this SOA was used in the NoGo trials) using a three-way repeated-measures ANOVA with factors Condition (compatible, incompatible, NoGo), Practice (first half, second half), and Laterality (left, right). We found a significant main effect of Condition, *F*_(2,18)_ = 5.56, *p* = 0.035. *Post hoc t*-tests revealed that the N1 amplitude was significantly larger for the NoGo condition (mean = −6.85, *SD* = 2.19 μV) compared to the compatible and incompatible conditions (mean = −5.88, *SD* = 2.12 μV) (*t*_(9)_ = 3.12, *p* = 0.012), whereas there was no difference between the compatible and incompatible Go conditions (*p* = 0.75; Figure 5).

#### P2

**Figure 6:**
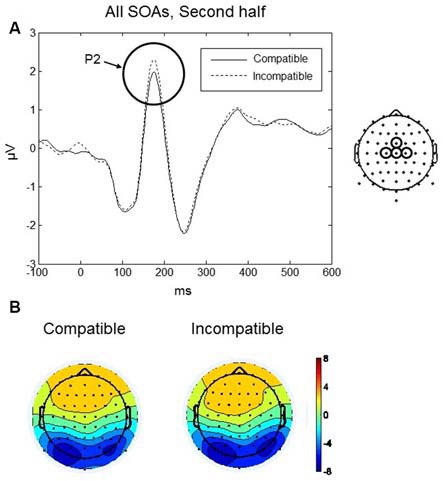
**Go-stimulus locked responses for the P2 component. (A)** ERPs at the central electrode cluster are plotted for compatible (solid line) and incompatible (dashed line) conditions, in the second half of the experiment, averaged across all SOAs. Between 140–200 ms, incompatible signals were more positive than compatible signals. **(B)** Scalp topographic maps at 170 ms after onset of the Go-stimulus for compatible (left) and incompatible (right) conditions.

The P2 component was largest over frontal scalp at around 170 ms (see Figures 6A,B). Mean amplitudes within fronto-central (AFz, Fz, F2, F1) and central (FCz, Cz, C1, C2) electrode clusters were analyzed in a 140–200 ms time window. A four-way repeated measures ANOVA with factors, Practice, SOA, and Region (fronto-central, frontal) revealed a significant interaction between Practice, Compatibility and Region, *F*_(1,9)_ = 8.13, *p* = 0.019. To further understand the interaction effect, a two-way repeated measures ANOVA was conducted with factors Compatibility and Region, separately for each level of the factor Practice (i.e., first and second halves of the experiment). No significant effects of Compatibility were found during the first half of the experiment. During the second half of the experiment, there was a significant interaction between Compatibility and Region, *F*_(1,9)_ = 7.14, *p* = 0.026. *Post hoc* paired *t*-tests revealed a significant difference between compatible and incompatible conditions at the central cluster, *t*_(9)_ = 2.785, *p* = 0.021. As shown in Figure 6A, the P2 amplitude was higher in the incompatible condition (mean = 1.56, *SD* = 1.42 μV) compared to the compatible condition (mean = 1.23, *SD* = 1.44 μV).

#### N2

**Figure 7:**
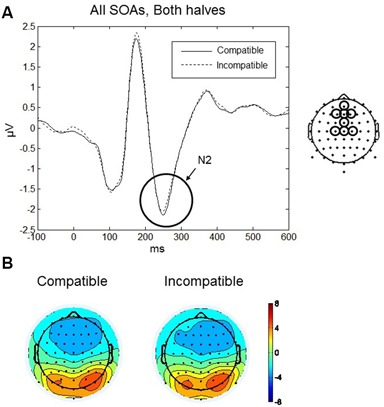
**Go-stimulus locked responses at N2. (A)** ERPs to compatible (solid line) and incompatible (dashed line) conditions, in both halves of the experiment, averaged across SOAs, are plotted for central and fronto-central regions. Between 220–280 ms, there was no difference between compatible and incompatible conditions. **(B)** Scalp topographic maps at 250 ms after onset of the Go-stimulus for compatible (left) and incompatible (right) conditions.

The maximum amplitude for N2 was observed at 250 ms, and the scalp distribution showed greatest negative amplitudes over the frontal and fronto-central scalp (Figures 7A,B). Mean amplitudes within the frontal and fronto-central clusters were analyzed between 220–280 ms. No main effects or interactions reached significance. Thus, in both halves of the experiment and for all SOAs no differences between compatible and incompatible conditions were found. Figure 7A shows the N2 component averaged over both halves of the experiment and all three SOAs for compatible and incompatible Go-stimuli.

### Lateralized Readiness Potentials

**Figure 8:**
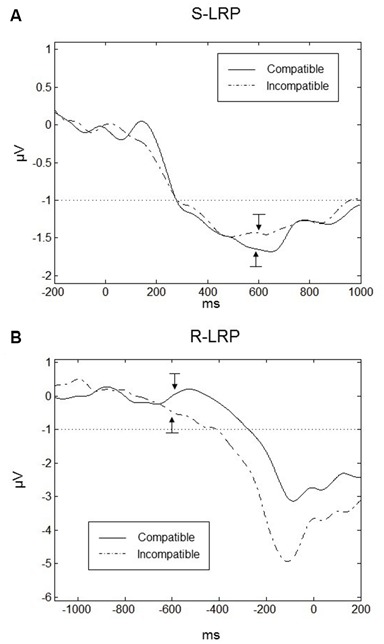
**Lateralized readiness potentials (LRPs). (A)** Go-stimulus locked LRPs are plotted 200 ms prior to the Go-stimulus up to 1000 ms following the Go-stimulus. There was no difference in S-LRP onset time between compatible (solid line) and incompatible (dashed line) conditions. The onset of the Go-stimulus occurred at 0 ms on the time scale. The arrows indicate the mean onset of the response (the RTs) for the compatible and incompatible trials. **(B)** Response-locked LRPs (R-LRP) for compatible and incompatible conditions. Onset of R-LRP was earlier for incompatible compared to compatible conditions. The onset of the response occurred at 0 ms on the time scale. The arrows indicate the mean onset of the Go-stimuli for compatible and incompatible trials. In both graphs, the dotted line in both graphs indicates the threshold for the determination of the LRP onsets and the bars related to the arrows correspond to ±1 standard error.

LRPs are important indicators of the preparation of the responses. In particular we were interested in the onset time of the LRPs under compatible and incompatible conditions. To obtain a sufficient number of trials, data had to be averaged over the SOAs. Grand averaged waveforms are displayed for Go-S-LRPs and R-LRPs in Figures 8A,B respectively. For S-LRPs a repeated-measures ANOVA with the factor Compatibility revealed no difference between compatible (mean onset = 278.5, *SD* = 26.0 ms) and incompatible (mean onset = 282.2, *SD* = 42.6 ms) conditions *F*_(1,9)_ = 0.096, *p* = 0.764.

For R-LRPs a repeated-measures ANOVA with the factor Compatibility revealed a significant difference between compatible and incompatible conditions *F*_(1,9)_ = 5.741, *p* < 0.05. Onset of the R-LRP in the incompatible conditions was earlier (mean = −410.2, *SD* = 75.6 ms) compared to R-LRP onset in the compatible condition (mean = −275.4, *SD* = 59.9 ms).

For R-LRPs we observed a difference in mean amplitude between conditions (*t*_(9)_ = 2.451, *p* = 0.037), where LRPs in the incompatible condition were more negative (Mean = −2.62, *SD* = 1.16 μV) compared to the compatible condition (Mean = −1.44, *SD* = 1.01 μV). For Go-stimulus locked LRPs there was no difference in mean amplitudes between compatible and incompatible conditions (*t*_(9)_ = 1.385, *p* = 0.020).

## Discussion

The present experiment explored the nature of effect anticipation during response preparation using an indirect priming paradigm. The experiment was a modified version of Experiment 3 from Ziessler et al. (2012), but here we additionally measured ERPs to examine differences in neural processing between effect compatible and effect incompatible Go-stimuli, and used longer SOAs to better disentangle ERPs to the Go-stimuli from ERPs to the imperative stimuli.

### Behavioral Data

The behavioral data confirmed the results from Ziessler et al.’s (2012) experiment: the compatibility between the Go-stimuli and the response effect significantly affected the RTs. Participants were instructed to prepare a response after presentation of the imperative stimulus, but to withhold the response until a Go-stimulus appeared. The significant reduction of the RTs with the increasing SOAs indicates that the participants indeed used the time for the preparation of the responses. Most importantly, as in the previous experiment, the RTs after the Go-stimulus depended on the relationship between the Go-stimulus and the stimulus that followed the execution of the response as a response effect. If the Go-stimulus was compatible with the response effect, RTs were shorter than in the incompatible condition. The Go-stimuli itself could not give any indication about the response. Each Go-stimulus was related to a response with a compatible and with an incompatible effect the same amount of times. Also, the comparison between compatible and incompatible conditions included exactly the same responses and the same Go-stimuli on both sides. What made the compatible and incompatible conditions different was the relationship between the Go-stimulus and a stimulus that is presented after the execution of the response as a response effect. Consequently, the difference between compatible and incompatible conditions can only be explained by the assumption that representations of the response effect were present when the Go-stimulus was processed. In other words, we have to conclude that when participants start to prepare a response they also anticipate the response effect. Only then the Go-stimuli can affect the RTs depending on their compatibility with the effect that will actually follow the response. To explain the compatibility effect we assume that each response preparation includes the anticipation of the effect. In the present experiment, during the anticipation process a stimulus (the Go-stimulus) is presented that is either compatible or incompatible with the effect. If the Go-stimulus is compatible with the response effect, this can prime the effect representation and facilitate the activation of the effect code in memory, whereas an incompatible Go-stimulus could inhibit or delay the activation of the effect code. As effect anticipation is part of response preparation this will finally affect the reaction time in the forced-choice task; the response will only be executed if there is an anticipation of the likely response effect (see also Ziessler et al., 2012).

Interestingly, for the first time we also found an interaction between the compatibility of the Go-stimuli with the response effects, the SOA between the imperative stimuli and the Go-stimuli, and practice. In the first half of the experiment compatibility effects were found at short and middle SOAs, in the second half of the experiment at middle and long SOAs. Thus, it seems that the participants in the early phases of the experiment activated response-effect representations at early phases of response preparation, probably already with response selection. This would be in line with strong versions of the ideomotor principle stating that response selection is based on effect anticipation (Elsner and Hommel, 2001; Hommel et al., 2001). However, in later phases of the experiment, i.e., with sufficient practice, the responses became more and more directly associated with the imperative stimuli that completely determined the responses in the forced-choice reaction task. In line with Herwig et al. (2007), it can be argued that participants switched from acting in an “intention mode” to acting in a “response mode”. Actually the response effects were completely irrelevant for the forced-choice task in the test phase. After learning the stimulus-response assignment during the first trials of the test phase, participants could prepare and execute their responses only driven by the colored squares. However, obviously they still anticipated the response effects for the selected response. But in the “response mode” this might happen after response selection, and not for the purpose of response selection as in the “intention mode”. Probably, in the “response mode”, the anticipated effects were used then for later processes such as response monitoring or error detection (Schmidt, 1975, 1988; Ziessler and Nattkemper, 2011).

### Event-Related Potential (ERP) Data

Additional evidence about the function of the effect codes was obtained from the ERP data, due to its excellent temporal resolution (on the level of milliseconds). In the current experiment we were mainly interested in the ERPs evoked by the Go-stimuli, and our analyses focused on the P1, N1, P2, and N2 components which were time-locked to the onset of the Go-stimuli. We also examined neural processes related to motor preparation using S-LRPs and R-LRPs.

In general, we only found ERP differences between compatible and incompatible Go-stimuli in the second half of the experiment. This is not in line with behavioral data in which we also found a compatibility effect in the first half of the experiment, in particular with 0 ms SAO. Probably the higher temporal variability at the beginning of the test phase led to a smearing effect that made it impossible to detect the ERPs. In fact, the reaction time data show a higher variance in the first half of the experiment for the shorter SOAs. In the first half the mean standard deviation of the reaction times for each participant and condition was about 176 ms as compared to 135 ms in the second half. Alternatively, participants might need sufficient practice with the task and sufficient time to anticipate the response effect before the Go-stimulus is presented. The rationale behind could be that differences in the processing of the Go-stimuli indicated by ERP differences can only appear if the effect codes are at least partially activated at the onset of the Go-stimulus.

#### P1

Differences between compatible and incompatible Go-stimuli were observed for the P1 component, which was maximal over bilateral occipito-parietal electrodes at around 110 ms after onset of the Go-stimuli. For effect-compatible Go-stimuli higher positive amplitudes were observed than for effect-incompatible Go-stimuli. The P1 is an early visual component that can be modulated by spatial attention; for example Van Voorhis and Hillyard (1977) found larger P1 amplitudes if the stimulus was presented in the attended visual field, and Mangun and Hillyard (1991) argued that the expectancy induced by the precuing of the target location would lead to a facilitation of the sensory-perceptual processing if the target was presented at the expected location. Taylor (2002) reported evidence that the P1 component is not only sensitive to spatial attention but also to a variety of non-spatial task demands. For example, in a visual search task the P1 amplitude to target stimuli (i.e., expected stimuli) was higher than the amplitude to non-targets. In line with those findings, Akyürek and Schubö (2013) found evidence for correlations between the P1 and early attentional feature selection. In summary, a wealth of evidence supports the claim that P1 reflects early processing that is modulated by top-down processing. Top-down processing prepares the sensory system for particular stimuli and enhances the processing of those stimuli if they are presented.

A difference between effect-compatible and incompatible Go-stimuli at the level of the P1 is a striking finding given the short latency of this component. We argue that the increased P1 amplitude for effect-compatible Go-stimuli can be interpreted as reflecting enhanced processing of the Go-stimulus if the Go-stimulus is compatible with the anticipated effect. For the 700 ms SOA it can be assumed that the response is fully prepared when the Go-stimulus appears. Furthermore, as discussed above, the behavioral data support the assumption that the preparation of the response included the anticipation of the response effect. Depending on the anticipated hand posture, the perceptual processing of the corresponding object is prepared, leading to the increased P1 amplitude for the compatible Go-stimuli. If this interpretation is correct, then it is not surprising that the P1 differences between compatible and incompatible Go-stimuli were only found in the second half of the experiment and only for the longest SOA. Participants needed sufficient practice and sufficient time to fully prepare the required response including the anticipation of the correct effect.

It is noteworthy that the P1 difference between effect-compatible and incompatible Go-stimuli was confined to the left occipito-parietal scalp region. While our experiment does not allow us to draw any firm conclusions regarding the lateralization of the P1 effect, nevertheless we may speculate that enhanced processing in the left visual cortex in the compatible condition may be due to the fact that the right side of the Go-stimulus object was more important for grasping purposes. Indeed the anticipated effects were right hands as viewed from the position of the participant (see Figure 1), and the Go-stimuli themselves all depicted objects that would be grasped from the right side (for instance, brush with the handle on the right etc.) Previous research has shown that the part of an object that is relevant for action (grasping) receives preferential allocation of attention (Brouwer et al., 2009), therefore it would fit that when the Go-stimulus is compatible with the anticipated effect, perceptual attention processes as indexed by the P1 would be enhanced in the visual cortex contralateral to the action-relevant (i.e., right) side of the Go-stimulus object. A future study could empirically test this explanation, for example by manipulating whether the anticipated effect depicted a left or a right hand, or whether the Go-stimulus object could be grasped by the left or right hand.

#### N1

The second ERP component we analyzed was the visual N1 which is an early visual component that is sensitive to selective attention (for review, see Mangun, 1995). According to Van Voorhis and Hillyard (1977), N1 modulation indicates the amplification of visual information at attended locations. However, the N1 is not only modulated by spatial attention, but also by object-based attention. N1 amplitudes evoked by attended objects were bigger than amplitudes evoked by unattended objects (Martínez et al., 2006). Vogel and Luck (2000) considered the visual N1 component as an indicator of a discrimination process, as the N1 is enhanced in conditions in which discrimination between classes of stimuli is required. Further, Antal et al. (2000) placed the N1 in the context of higher-level categorization processes. In the present experiment, we did not find any difference in the N1 following compatible and incompatible Go-stimuli. All stimuli were presented at the expected location in the middle of the screen and all objects presented as Go-stimuli had the same frequency. Therefore, no difference in the N1 component should be expected between compatible and incompatible conditions.

However, if the NoGo condition is considered as well, participants needed to discriminate in each trial between the category of the Go-stimuli (i.e., coffee mug, computer mouse, pen and spoon) and the NoGo stimulus (i.e., hammer). In the majority of trials, the presented object belonged to the category of the Go-stimuli. In the remaining trials the hammer as a NoGo stimulus had to be discriminated from the Go-stimuli. Our analyses indeed revealed larger N1 amplitudes for the NoGo stimuli compared to the Go-stimuli, reflecting discrimination between the task-relevant categories of Go vs. NoGo signals.

#### P2

The third ERP component we analyzed was the P2, maximal over fronto-central scalp at around 170 ms following onset of the Go-signal. The P2 is assumed to reflect processes of visual feature detection and analysis (Hillyard and Münte, 1984). Luck and Hillyard (1994) considered the P2 as part of a cognitive matching system that compares sensory input with expectations derived from memory. Larger P2 amplitudes are observed if stimuli violate the expectations in a given context (Ferretti et al., 2008), as the detection of anomalies should lead to more extensive processes of feature detection and analysis (Bohan et al., 2012). In fact, this view fits very well with the P2 effects observed in the present experiment. In incompatible trials the P2 amplitude was larger compared to the compatible trials. This effect was found in the second half of the experiment independently of the SOA between the imperative stimulus and the Go-stimulus.

Our interpretation is quite similar to the interpretation of the P1 difference between compatible and incompatible trials. After sufficient practice with the task the participants anticipated the response effect as soon as the response was selected. The effect representation tuned the cognitive system to process a compatible stimulus. This led to higher P1 amplitudes after compatible Go-stimuli, but also to higher P2 amplitudes after incompatible Go-stimuli that did not match with the activated representations of the response effect. Thus, if the Go-stimuli are incompatible with the effect, more extensive feature processing seems to be necessary.

#### N2

The N2 component of the Go-stimulus locked ERPs was prominent over fronto-central areas at a peak latency of around 250 ms. The N2 is related to target frequency in visual search tasks (Luck and Hillyard, 1990), and in flanker tasks (Eriksen and Eriksen, 1974) the fronto-central N2 correlated with conflict detection and conflict monitoring (Donkers and van Boxtel, 2004; Purmann et al., 2011; Leue et al., 2012). In the present experiment, target frequency could not lead to differences in the N2 component between the compatible and incompatible conditions because all Go-stimuli had exactly the same frequency and the frequency of the NoGo stimuli (which were not involved in the N2 analysis) was only slightly smaller.

A more interesting question is why we did not find evidence of conflict detection or monitoring in the incompatible trials. Usually in the flanker paradigm larger N2 amplitudes are observed for incompatible trials (e.g., Purmann et al., 2011). However, that seems to be related to the conflict between the target and the flanker stimuli that activate two different responses, as the flanker related response needs to be inhibited. Related to Go/NoGo, Folstein and van Petten (2008) described the N2 as indicator of a control mechanism for response inhibition. The fact that no N2 difference was found between compatible and incompatible Go trials in the present experiment may indicate that the incompatible Go-stimuli did not lead to a response conflict. This confirms the conclusion by Ziessler et al. (2012) that compatible Go-stimuli led to a facilitation of the response; but incompatible Go-stimuli did not inhibit the response. The facilitation in compatible trials seemed to be based on faster processing of the Go-stimuli that were compatible with the activated memory representation of the response effect (c.f. the larger P1 amplitude) and the extended processing of Go-stimuli that were incompatible with the effect representation (c.f. the larger P2 amplitude). In both cases it was the top-down modulation of the visual processing of the Go-stimuli that was evoked by the anticipation of the response effect.

### LRPs

Finally, we analyzed the LRPs (Gratton et al., 1988; Coles et al., 1996). Whereas the P1, N1, P2, and N2 components of the Go-stimulus locked ERPs are related to visual processing and stimulus evaluation, the LRP reflects the selection and execution of motor responses. The onset latency of the Go-stimulus locked LRPs reflects the duration of the pre-motoric processes, and the onset latency of the R-LRPs indicates the duration of the motoric processes (Leuthold, 2003). Interestingly, in our experiment there was no difference in the onset of the Go-stimulus locked LRPs, but only in the R-LRPs. In the incompatible condition the time between the onset of the LRPs and the execution of the response was longer than in the compatible condition. In other words, at a time at which the response was fully prepared, the execution of the response was delayed if the Go-stimulus was incompatible with the effect of the prepared response. We can only speculate about the reasons for the delayed execution of the response. A plausible explanation is that a Go-stimulus that was incompatible with the response effect reduced the activation of the effect representation. This may have led to uncertainty about the actual effect and consequently affected the monitoring of the response execution.

For R-LRPs we observed that amplitudes were more negative in the incompatible condition compared to the compatible condition. We speculate that the reduced LRP amplitude indicated that fewer resources were necessary for motor processes when the Go-stimulus was compatible with the effect of the prepared response. On the hand other, increased neural processing was required in the incompatible condition, suggestive of less efficient motor-related processing. There were no differences in amplitude for the S-LRPs, indicating that the compatibility of the Go-stimuli did not affect pre-motor stages of processing.

## Conclusions

In sum, the behavioral data provided clear evidence for the activation of effect codes during the preparation of the responses. Otherwise it would be difficult to explain why the Go-stimuli would affect the responses. It is only the relationship to the learned response effects that makes the difference between compatible and incompatible and incompatible Go-stimuli. The analysis of the ERPs provided important additional evidence to understand the neural processes linked with the anticipation of the response effects.

The difference in the early visual P1 between compatible and incompatible trials shows that very early in the preparation of the response the sensory system is tuned to perceive the predicted response effect. In line with Roussel et al. (2013), the P1 difference supports the notion that effect anticipation consists in the internal pre-activation of the sensory representation of the expected effects (see also Hughes and Waszak, 2011). The increased amplitudes of the frontal P2 for incompatible trials reflected the mismatch between the anticipated response effects and the incompatible Go-signal. Finally, the extended R-LRPs in incompatible trials indicated prolonged motoric processes to execute the response. This may be related to difficulties in the monitoring of response execution due to the conflict between the Go-stimulus and the anticipated response effect.

In summary, the ERP and LRP data support the view that effect anticipation contributed to the preparation of motor responses at all levels including perceptual, cognitive and motor components. The anticipation of response effects does not only lead to sensory attenuation. Representations of the effects are activated in memory and those representations are available for the cognitive processes of response preparation. Moreover, the early visual ERPs provided evidence for a sensory representation that can directly be related to the stimuli from the external world. The R-LRP differences showed that the effect codes were also involved in response execution.

## Author Contributions

NRH acquired the data and analyzed the EEG data, and contributed to writing the manuscript. MZ designed the study, analyzed the behavioral data, and contributed to writing the manuscript.

## Conflict of Interest Statement

The authors declare that the research was conducted in the absence of any commercial or financial relationships that could be construed as a potential conflict of interest.
